# HDV Seroprevalence in HBsAg-Positive Patients in China Occurs in Hotspots and Is Not Associated with HCV Mono-Infection

**DOI:** 10.3390/v13091799

**Published:** 2021-09-10

**Authors:** Imme Roggenbach, Xiumei Chi, Florian A. Lempp, Bingqian Qu, Lisa Walter, Ruihong Wu, Xiuzhu Gao, Paul Schnitzler, Yanhua Ding, Stephan Urban, Junqi Niu

**Affiliations:** 1 Department of Hepatology, First Hospital of Jilin University, Changchun 130021, China; imme.roggenbach@gmx.de (I.R.); chixm@jlu.edu.cn (X.C.); wuruihong@jlu.edu.cn (R.W.); xiuzhugao@jlu.edu.cn (X.G.); 2 Department of Infectious Diseases, Molecular Virology, University Hospital Heidelberg, 69120 Heidelberg, Germany; f.lempp@gmx.net (F.A.L.); qubingqian@gmail.com (B.Q.); lisa.walter@med.uni-heidelberg.de (L.W.); 3 Phase I Clinical Trials Unit, First Hospital of Jilin University, Changchun 130021, China; dingyanh@jlu.edu.cn; 4 Department of Infectious Diseases, Virology, University Hospital, 69120 Heidelberg, Germany; paul.schnitzler@med.uni-heidelberg.de

**Keywords:** Hepatitis Delta virus, seroprevalence, epidemiology, HBsAg, helper virus, Hepatitis C virus, intravenous drug use, cirrhosis, China, Germany

## Abstract

HDV infection causes severe liver disease, the global health burden of which may be underestimated due to limited epidemiological data. HDV depends on HBV for infection, but recent studies indicated that dissemination can also be supported by other helper viruses such as HCV. We used a rapid point-of-care test and an ELISA to retrospectively test for antibodies against the Hepatitis Delta antigen (anti-HDV-Ab) in 4103 HBsAg-positive and 1661 HBsAg-negative, anti-HCV-positive sera from China and Germany. We found that the HDV seroprevalence in HBsAg-positive patients in China is limited to geographic hotspots (Inner Mongolia: 35/251, 13.9%; Xinjiang: 7/180, 3.9%) and high-risk intravenous drug users (HBV mono-infected: 23/247, 9.3%; HBV-HCV co-infected: 34/107, 31.8%), while none of the 2634 HBsAg carriers from other metropolitan regions were anti-HDV-Ab-positive. In Germany, we recorded an HDV seroprevalence of 5.3% in a university hospital environment. In a cohort of HBsAg-negative, anti-HCV-positive patients that were not exposed to HBV before (anti-HBc-negative), HDV was not associated with HCV mono-infection (Chinese high-risk cohort: 0/365, 0.0%; German mixed cohort: 0/263, 0.0%). However, 21/1033 (2.0%) high-risk HCV patients in China with markers of a previously cleared HBV infection (anti-HBc-positive) were positive for anti-HDV-Ab, with two of them being positive for both HDV and HCV RNA but negative for HBV DNA. The absence of anti-HDV-Ab in HCV mono-infected patients shows that HCV cannot promote HDV transmission in humans.

## 1. Introduction

Hepatitis Delta virus (HDV) is the causative agent of the most severe form of viral liver disease, affecting more than 12 million people worldwide. As a satellite virus, HDV depends on the helper function of the Hepatitis B virus (HBV) that provides its envelope proteins (HBsAg) for receptor-mediated entry into host cells [1,2,3]. HDV transmission occurs parenterally via co-infection with HBV or the super-infection of chronically infected HBV patients. The main transmission routes include needle sharing during intravenous drug use (IVDU), high-risk sexual behavior, and the exchange of body fluids in private households and low-hygiene communities [4,5]. Since HDV infection depends on the presence of HBV, the EASL, AASLD, and APASL guidelines recommend testing for HDV in all HBsAg-positive, chronically infected HBV patients [6,7,8]. However, these guidelines are not strictly followed in the clinical routine [9]. Reasons may comprise insufficient access to testing equipment and the low awareness for HDV among healthcare providers. Moreover, the lack of effective therapeutic options until recently contributed to limited testing efforts. As this has now changed with the recent approval of the first HDV antiviral drug Bulevirtide (Hepcludex) in Russia and the European Union [10], the rapid and reliable identification of infected individuals eligible for treatment becomes an important issue [11,12,13]. HDV is diagnosed in a two-step procedure, starting with the detection of antibodies against the Hepatitis Delta antigen (anti-HDV-Ab) by immunoassay, followed by the quantification of HDVA RNA by qPCR. IgM antibodies are markers for an early-stage acute infection, whereas IgG appears later but persists upon chronification or HDV RNA clearance [14]. Most diagnostic immunoassays for HDV focus on the detection of IgG due to the higher longevity of the response.

The World Health Organization estimates that 5% of HBsAg carriers are co-infected with HDV worldwide. However, different meta-studies state different estimates on the global HDV prevalence and for several countries, accurate epidemiological data about HDV is entirely lacking [4,15,16,17]. High HDV endemicity was recorded in Mongolia, where over 50% of HBsAg carriers are positive for anti-HDV-Ab [18,19]. A high seroprevalence in HBsAg carriers of over 15% was also observed in the Amazon basin [20], in Central Asia [21], and in some African countries [22]. In China, a recent meta-analysis concluded that 5.6% of a mixed HBsAg-positive population is co-infected with HDV, while the seroprevalence is significantly higher in IVDU with 60.7% [4]. However, other reports on the prevalence of HDV in China are contradictory. While one study showed an HDV seroprevalence of 1–2% across the whole country [23], other multi-center studies indicated that HDV highly accumulates in autonomous regions [24,25,26]. Therefore, it currently remains unclear whether HDV is evenly distributed in China or occurs in hotspots.

Current knowledge about HDV assumes that it exclusively associates with markers of an HBV infection. Yet, recent experimental evidence suggested that other viruses (e.g., HCV, VSV) can support HDV envelopment and dissemination in vitro and in vivo [27]. In particular, it was shown that the HDV ribonucleoprotein complex can be packaged in envelope proteins of the Hepatitis C virus (HCV) to form infectious particles. These particles entered Huh-106 target cells in a CD81-receptor-dependent manner. Moreover, HCV-pseudotyped HDV was able to initiate HDV infection and propagate in liver-humanized immunodeficient Fah^−/−^/Rag2^−/−^/Il2rg^−/−^ mice. This gave rise to the hypothesis that HDV may also exploit HCV to disseminate in humans. Furthermore, HDV-like RNA sequences were recently found in snakes [28], ducks [29], amphibians, fish, and termites [30] in the absence of a respective hepadnaviral helper virus, although most of these HDV-like genomes do not encode a large HDAg or a prenylation site, both prerequisites for envelopment with hepadnaviral proteins. Only one clinical case of HBV-independent HCV-HDV co-infection with positive HDV RNA has been reported in humans so far [31]. Since two other studies with more than 2000 patients did not identify HCV-supported HDV infection in the absence of HBV [32,33], the role of HCV needs to be further evaluated in populations where HCV, HBV, and HDV are highly endemic.

In this study, we retrospectively evaluated the seroprevalence of HDV in HBsAg-positive and HBsAg-negative populations in China and Germany using a rapid point-of-care (POC) device to pan-genotypically detect anti-HDV-Ab in serum samples. We show that HDV is not common in HBsAg-positive patients from metropolitan hospitals in China but is present in geographic hotspots and a high-risk population. HDV replication did not occur in needle-sharing HCV mono-infected intravenous drug users in the absence of a previous or ongoing HBV infection.

## 2. Materials and Methods

### 2.1. Study Population

A total of 5080 patient sera were retrospectively analyzed for the seroprevalence of anti-HDV-Ab. A total of 3065 sera were collected from HBsAg-positive patients previously admitted to multiple Chinese hospitals in seven provinces (Jilin, Hubei, Jiangsu, Liaoning, Guangdong, Shandong, and Heilongjiang), three autonomous regions (Inner Mongolia, Xinjiang, and Tibet), and one municipality (Shanghai) in 2019. Additionally, HBsAg-positive patients that were either HBV mono-infected (247 sera) or HBV-HCV co-infected (107 sera) and HBsAg-negative HCV mono-infected patients (anti-HBc-positive: 1033 sera; anti-HBc-negative: 365 sera) from the remote Chinese town Fuyu (Jilin province) were tested. The sera of these patients were characterized for HBV and HCV during previous epidemiological studies in 2012 and 2017, as Fuyu is known for abundant needle sharing during IVDU [34]. Samples from 684 HBsAg-positive patients and 263 HBsAg- and anti-HBc-negative HCV patients admitted to the University Hospital Heidelberg (Germany) between 2014 and 2019 were also included. The 251 HBsAg-positive individuals from Inner Mongolia were categorized according to their state of liver disease using standard diagnostic criteria (e.g., irregularities in liver biopsies and medical scans, elevated ALT levels, etc.). For 21 patients, the state of liver disease was not defined due to a lack of medical data. Ethical approvals for this study were obtained from both the ethical commission at the University Hospital Heidelberg (Germany) and the First Hospital of Jilin University (China).

### 2.2. Serological Tests

Chinese patient sera and HBsAg-positive sera from Heidelberg (Germany) were tested for anti-HDV-Ab (IgG) using a pan-genotypic lateral flow assay (HDV rapid test), as previously described [35]. The assay runs on 10 µL of patient serum per test and is read out visually after 20 min. Compared to a commercial ELISA, the HDV rapid test has a sensitivity of 94.6% and a specificity of 100%. In Germany, HBsAg- and anti-HBc-negative samples from HCV patients were tested with an in-house ELISA [36] using the same recombinant Hepatitis Delta antigen (HDAg). Samples from the clinical routine in Heidelberg were tested for HBsAg, anti-HBc, and anti-HCV using the ADVIA Centaur^®^ HBsAgII, aHBcM, and aHCV tests (Siemens Healthineers, Erlangen, Germany), respectively. In China, HBsAg and anti-HBc screening was performed with the ELISA HBV test (Shanghai Kehua Bio, Shanghai, China). HBsAg-positive results were confirmed with the Elecsys^®^ HBsAg II quant II kit on the cobas e 411 analyzer (Roche, Basel, Switzerland). Anti-HCV was detected with the ELISA HCV test (Shanghai Kehua Bio, Shanghai, China).

### 2.3. Qualitative Anti-HDV-Ab ELISA

*E. coli*-derived recombinant HDAg (2 mg/mL) with a non-naturally occurring consensus sequence of all eight HDV genotypes (patent no. WO2019219840A1) was diluted 1:2,000 in coating buffer (13 mM Na_2_CO_3_, 88 mM NaHCO_3_, pH 9.2) to a final concentration of 1 μg/mL. ELISA plates were coated with 50 ng (50 μL) of diluted recombinant HDAg overnight at 4 °C. Plates were washed 1× with 200 μL of washing buffer (0.05% Tween^®^ 20 *v*/*v* in PBS) and blocked with 200 μL of blocking buffer (1% casein sodium salt *w*/*v*, 0.05% Tween^®^ 20 *v*/*v* in PBS) per well for 1 h at room temperature. Serum dilutions were prepared in dilution buffer (0.1% casein sodium salt *w*/*v*, 0.05% Tween^®^ 20 *v*/*v* in PBS). To confirm anti-HDV-Ab-positive results from the HDV rapid test, patient sera were serially diluted 1:8 from an initial dilution of 1:100. After blocking, plates were washed 1× with 200 μL of washing buffer and 50 μL of sample dilutions were added. Binding of anti-HDV-Ab to HDAg was performed for 1 h at 37 °C. Plates were washed 5× with 200 μL of washing buffer. Secondary HRP-conjugated goat anti-human IgA/IgG/IgM (109-035-064, Jackson Immunoresearch, West Grove, PA, USA) was diluted 1:10,000 in dilution buffer in a total volume of 100 μL per well and incubated in the plates for 1 h at 37 °C. Plates were washed 5× with 200 μL of washing buffer. The detectable product was generated by the addition of 100 μL of TMB substrate (00-4201-56, eBioscience, San Diego, CA, USA) per well and HRP-mediated decay was allowed to take place for 10 min in the dark. Reactions were stopped with 100 μL of stop solution (1 M H_3_PO_4_) per well and the OD450 was quantified using the EnVision^TM^ Multilabel Reader (2101–0010, Perkin Elmer, Waltham, MA, USA).

### 2.4. Quantification of Virus Load

HDV RNA from anti-HDV-Ab-positive samples was quantified to confirm active HDV replication. RNA was extracted from >400 µL serum using the INSTANT Virus RNA/DNA Kit (Analytik Jena, Jena, Germany) and the RNA was quantified with the RoboGene HDV RNA Quantification Kit 2.0 (RoboScreen Diagnostics, Leipzig, Germany) on the 7500 Fast instrument (Applied Biosystems, Foster City, CA, USA). The limit of detection as specified by the manufacturer was 6 IU/mL. HBV DNA and HCV RNA were quantified using the COBAS AmpliPrep/COBAS TaqMan HBV and HCV tests, respectively (Roche, Basel, Switzerland).

### 2.5. RT-PCR, PCR, and Nested PCR

Purified viral RNA from patient serum was reverse transcribed using the SuperScript IV Reverse Transcriptase (RTase) (18090050, Invitrogen, Carlsbad, CA, USA). An amount of 5 µL of viral RNA was mixed with 0.8 µL of dNTP, 2 µL of random primers (4368813, Thermo Fisher Scientific, Waltham, MA, USA), and 5.2 µL of nuclease-free water. The mix was incubated for 5 min at 65 °C and chilled on ice for 2 min. For RT-PCR, 4 µL of SSIV buffer, 1 µL of DTT (100 mM), 1 µL of RNase inhibitor, and 1 µL of SuperScript IV RTase (200 U/µL) were added and the RT-PCR was run for 10 min at 23 °C, 20 min at 50 °C, and 10 min at 80 °C. One-step PCR was performed using the following PCR mix: 2µL of RT-PCR product, 5 µL of PCR buffer, 2.5 µL of MgCl_2_ (25 mM), 0.5 µL of dNTP (10 mM), 0.5 µL of primer p890 (10 µM, 5′-CATGCCGACCCGAAGAGGAAAG-3′), 0.5 µL of primer p1265 (10 µM, 5′-GAAGGAAGGCCCTCGAGAACAAGA-3′) [37], 0.25 µL of GoTaq Flexi DNA polymerase (5 U/µL) (M8296, Promega, Madison, WI, USA), and 14 µL of nuclease-free water. The reaction was run for 5 min at 95 °C; 36 cycles of 30 s at 95 °C, 30 s at 42 °C, 1 min at 72 °C; and for 5 min at 72 °C. PCR products were purified from 2.5% agarose gels. For nested PCR, 5 µL of purified product from the first PCR was further amplified using p928 (5′-AACCCGTGAGTGGAAACCCGCT-3′) and p1210 (5′-GCAGGGAAGAAGAAGAGGAACT-3′) primers and the same PCR program.

### 2.6. Sanger Sequencing, Sequence Alignment, and Phylogenetic Analysis

Amounts of 5 µL of PCR-amplified and purified viral DNA were mixed with the respective primer pairs and sent for Sanger sequencing (Eurofins GATC, Luxemburg). The resulting sequences were assembled and manually corrected at both ends. A multiple sequence alignment was performed using the T-coffee platform (http://tcoffee.crg.cat/apps/tcoffee/do:regular, accessed on 8 September 2021) and BoxShade server (http://embnet.vital-it.ch/software/BOX_form.html, accessed on 8 September 2021). Patient sequences along with HDV reference sequences representing genotypes 1, 2, and 4 were used to construct a phylogenetic tree in MEGA 6 using a neighbor-joining algorithm with 1000 bootstrap replications [38].

### 2.7. Statistics

Confidence intervals (CIs) of 95% of HDV seroprevalences were calculated using the Wilson score interval. CIs were displayed in forest plots generated in GraphPad Prism 8. For statistical testing, a Chi-squared test was performed. Seroprevalences were considered to be significantly different when the Chi-squared test returned a value <0.05.

## 3. Results

### 3.1. HDV Occurs in Geographic Hotspots in China

**Figure 1:**
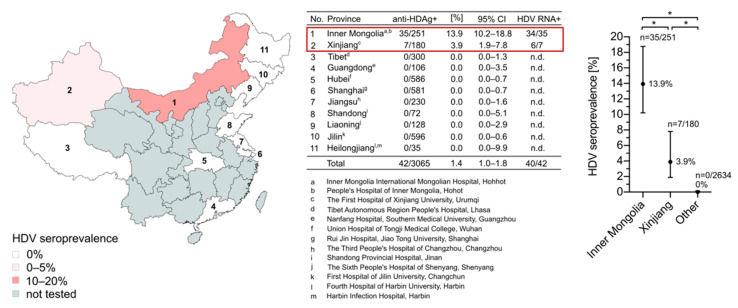
Geographic distribution of the HDV seroprevalence in China. Serum samples were collected from HBsAg-positive patients living in seven provinces (Liaoning, Shandong, Jilin, Heilongjiang, Guangdong, Jiangsu, and Hubei), three autonomous regions (Inner Mongolia, Xinjiang, and Tibet), and one municipality (Shanghai). All sera were tested for anti-HDV-Ab using the HDV rapid test. CIs of 95% were calculated according to the Wilson score interval and seroprevalences were compared using the Chi-squared test. * corresponds to a *p*-value < 0.05.

As a first aim of this study, we evaluated the HDV seroprevalence in China. We tested 3065 HBsAg-positive patient sera from multiple Chinese metropolitan hospitals for anti-HDV-Ab using the recently developed HDV rapid test [35]. Serum samples from seven provinces (Liaoning, Shandong, Jilin, Heilongjiang, Guangdong, Jiangsu, and Hubei), three autonomous regions (Inner Mongolia, Xinjiang, and Tibet), and one municipality (Shanghai) were included. Out of the 3065 samples, 42 were positive for anti-HDV-Ab (Figure 1). Remarkably, all positive samples originated from patients living in Inner Mongolia 13.9% (35/251, 95% CI: 10.2–18.8%) and Xinjiang 3.9% (7/180, 95% CI: 1.9–7.8%). The remaining 2634 samples collected from other sites were anti-HDV-Ab-negative (0.0%, 95% CI: 0.0–0.2%). These results indicate that HDV is unevenly distributed among HBsAg carriers in China and occurs in geographic hotspots.

### 3.2. HDV Seroprevalence Is Increased in Cirrhotic Patients from Inner Mongolia

**Figure 2:**
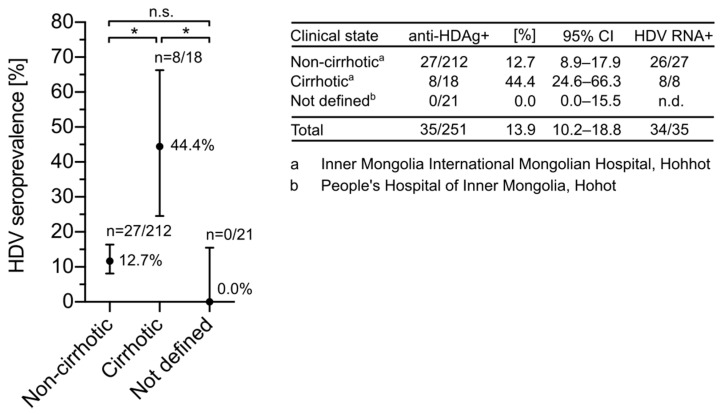
Distribution of HDV seroprevalence in Chinese patients at different stages of liver disease. Serum samples were collected from HBsAg-positive patients living in Inner Mongolia. The stage of liver disease was determined using standard diagnostic criteria. All sera were tested for anti-HDV-Ab using the HDV rapid test. CIs of 95%were calculated according to the Wilson score interval and seroprevalences were compared using the Chi-squared test. * corresponds to a *p*-value < 0.05.

HDV infection accelerates liver disease progression in HBsAg carriers, resulting in an earlier onset of cirrhosis and hepatocellular carcinoma [39,40]. Having access to pathological data from patients in Inner Mongolia, we assessed the seroprevalence of HDV in cirrhotic and non-cirrhotic patients (Figure 2). Consistent with previous findings [41], anti-HDV-Abs were more prevalent in cirrhotic compared to non-cirrhotic patients. While 44.4% (8/18, 95% CI: 24.6–66.3%) of cirrhotic patients showed serological markers for HDV infection, only 12.7% (27/212, 95% CI: 8.9–17.9) of sera from non-cirrhotic patients were anti-HDV-Ab-positive. These results confirmed the impact of HDV on the development of cirrhosis.

### 3.3. HDV Associates with HBsAg-Positive but Not with HBsAg-Negative, Anti-HBc-Negative HCV Patients from Germany

**Figure 3:**
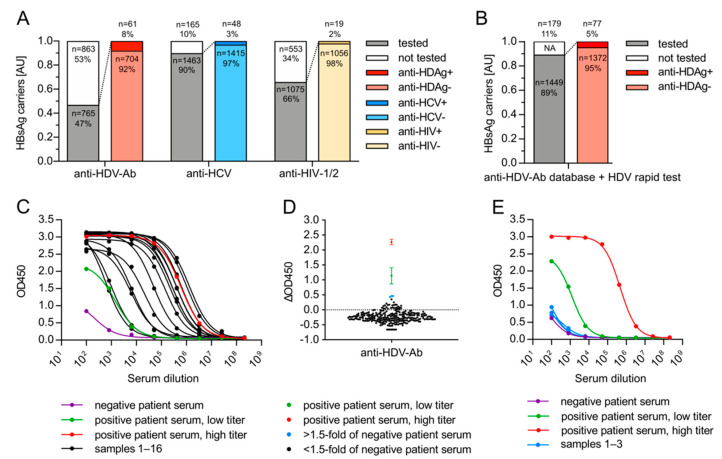
HDV seroprevalence in Germany. (**A**) Databank research of HBsAg-positive patients. Diagnostic testing data from 1628 HBsAg-positive patients that were admitted to the University Hospital Heidelberg between 2014 and 2019 were evaluated to determine the anti-HDV-Ab, anti-HCV, and anti-HIV 1/2 testing rates. (**B**) Testing for anti-HDV-Ab in HBsAg-positive patients. A total of 684 sera from HBsAg carriers admitted to the University Hospital Heidelberg between 2014 and 2019 with uncharacterized anti-HDV-Ab status were retrospectively tested for anti-HDV-Ab using the HDV rapid test. NA: samples for which no databank serum was available. (**C**) Validation of anti-HDV-Ab positivity. Sera that tested anti-HDV-Ab-positive using the HDV rapid test were re-tested using a standard, semi-quantitative in-house ELISA. Averaged results from three independent experiments are shown. (**D**) Testing for anti-HDV-Ab in 263 HBsAg-negative, anti-HBc-negative, anti-HCV-positive patients that were admitted to the University Hospital Heidelberg in 2019. Sera were tested for anti-HDV-Ab using an in-house ELISA. The averages of triplicate results at 1:100 dilutions are shown. Blue: sera showed equivocal ELISA signals >1.5-fold of the negative patient serum. (**E**) Re-evaluation of patient sera with equivocal anti-HDV-Ab testing results using a semi-quantitative in-house ELISA.

To determine the HDV seroprevalence in a representative university hospital environment in Germany, we performed a database analysis of 1628 HBsAg-positive patients admitted to the University Hospital Heidelberg (Germany) between 2014 and 2019 (Figure 3A). We found that only 47% (765/1628) of HBsAg-positive patients were tested for anti-HDV-Ab in the clinical routine, while for 53% (863/1628), the HDV status remained unknown. Remarkably, within the same group of HBsAg-positive patients, 90% were tested for anti-HCV (1463/1628) and 66% for anti-HIV (1075/1628), even though HCV-HBV (3.3%, 48/1463, 95% CI: 2.5–4.3%) and HIV-HBV (1.8%, 19/1075, 95% CI: 1.1–2.7%) co-infection rates were generally lower compared to HDV-HBV co-infection (8.0%, 61/765, 95% CI: 6.3–10.1%). We then tested the respective serum bank samples of 684 HBsAg-positive individuals that did not undergo HDV follow-up testing before using the HDV rapid test (Figure 3B) and identified 16 additional anti-HDV-Ab-positive patients. Combining these results with the databank analysis, a total of 77/1449 HBsAg-positive patients were anti-HDV-Ab-positive (5.3%, 95% CI: 4.3–6.6%) at the University Hospital Heidelberg between 2014 and 2019. We confirmed the reliability of the HDV rapid test results in all anti-HDV-Ab-positive sera using a standard, semi-quantitative in-house ELISA (Figure 3C).

So far, only one study identified a single patient serum that was positive for both HDV and HCV RNA in the absence of HBV markers (HBsAg and anti-HBc-negative) [31]. Two other studies did not find evidence for HCV-supported HDV infection in >2000 sera [32,33]. To investigate whether HCV-supported HDV infection can occur in humans, we tested for the presence of anti-HDV-Ab in 263 HBsAg-negative and anti-HBc-negative sera from anti-HCV-positive patients admitted to the University Hospital Heidelberg in 2019. Using our in-house ELISA, we detected elevated anti-HDV-Ab signals in 3/263 samples (Figure 3D, blue data points). However, these signals could not be confirmed by the HDV rapid test (data not shown). Furthermore, signals obtained from serial dilutions in a follow-up in-house ELISA were comparable to the negative control curve, indicating that unspecific binding of serum components led to elevated responses in the initial screen (Figure 3E). We therefore judged these samples as anti-HDV-Ab-negative. The comparative results of three different assays thus indicate that HCV-supported HDV infection in the absence of HBV is unlikely to occur in humans (0.0%, 0/263, 95% CI: 0.0–1.4%) and that caution should be taken in the choice of the assay.

### 3.4. HDV Is Highly Endemic in Patients with Positive HBV Markers from a High-Risk Needle-Sharing IVDU Population in China and Is Not Associated with HCV Mono-Infection

**Figure 4:**
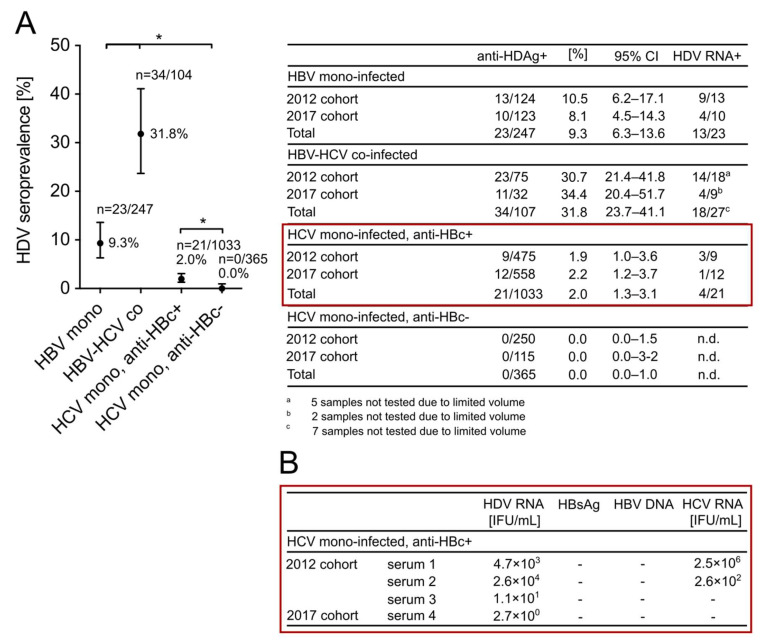
HDV seroprevalence in a Chinese high-risk population (Fuyu, Jilin province) known for abundant needle sharing during IVDU. Serum samples were collected in 2012 and 2017 and pre-characterized for HBsAg and anti-HCV during previous epidemiological studies conducted at the First Hospital of Jilin University (Jilin province, China). (**A**) Serological analysis for anti-HDV-Ab in Fuyu. Sera were retrospectively tested for anti-HDV-Ab using the HDV rapid test. Active HDV RNA replication was confirmed using the INSTANT Virus RNA/DNA Kit (Analyik Jena, Jena, Germany) for RNA extraction and the RoboGene HDV RNA Quantification Kit 2.0 (RoboScreen, Leipzig, Germany) on the 7500 Fast Instrument (Applied Biosystems, Foster City, USA) for RNA quantification. (**B**) Further characterization of HDV RNA-positive sera in the HBsAg-negative, anti-HBc-positive, anti-HCV-positive cohort. Sera were tested for quantitative HBsAg (Elecsys^®^ HBsAg II quant II kit, Roche, Basel, Switzerland), HBV DNA, and HCV RNA (COBAS AmpliPrep/COBAS TaqMan HBV and HCV Tests, Roche, Basel, Switzerland). CIs of 95% were calculated according to the Wilson score interval and seroprevalences were compared using the Chi-squared test. * corresponds to a *p*-value < 0.05.

A second aim of this study was to unambiguously clarify a possible helper function of HCV for HDV dissemination in humans. We analyzed serum samples from a previously identified high-risk population in the northeastern Chinese town Fuyu, in which HBV and HCV are highly endemic due to abundant needle sharing during IVDU. We retrospectively screened the sera of this population for anti-HDV-Ab and found a seroprevalence of 31.8% (34/107, 95% CI: 21.7–43.1%) in HBV-HCV co-infected patients (HBsAg-positive, anti-HCV-positive) and 9.3% (23/247, 95% CI: 6.3–13.6%) in HBV mono-infected patients (HBsAg-positive, anti-HCV-negative). These results indicated that HDV is endemic in Fuyu and has presumably been spread via a similar transmission route as has been described for HCV [34]. We then tested HCV mono-infected patients (anti-HCV-positive, HBsAg-negative) of this cohort for anti-HDV-Ab and differentiated those patients that were anti-HBc-positive or anti-HBc-negative. Interestingly, all HCV patients that lacked markers for an HBV infection (HBsAg and anti-HBc-negative) were negative for anti-HDV-Ab (0/365, 0%, 95% CI: 0.0–1.0%) (Figure 4A) compared to 21/1033 (2.0%, 95% CI: 1.3–3.1%) HCV patients from the anti-HBc-positive cohort. Remarkably, 4/21 of these patients were also positive for HDV RNA, yet with titers <1 × 10^5^ IU/mL (Figure 4B). Using more sensitive quantitative assays, we re-confirmed the absence of HBsAg and HBV DNA in these sera and tested for HCV RNA. Active HCV replication was detected in 2/4 samples that also showed the highest HDV serum RNA levels among the four HDV RNA-positive sera (serum 1: 4.7 × 10^3^ IU/mL HDV RNA and 2.5 × 10^6^ IU/mL HCV RNA; serum 2: 2.6 × 10^4^ IU/mL HDV RNA and 2.6 × 10^2^ IU/mL HCV RNA). We conclude that HDV transmission is strictly associated with the presence of HBV, while low HDV replication can occur in HDV-HBV-HCV triple-infected patients after HBV clearance.

### 3.5. HDV Genotype 1 Is Most Abundant in Fuyu and Forms a Cluster of Strains but HDV Genotype 2a Is Also Present

**Figure 5:**
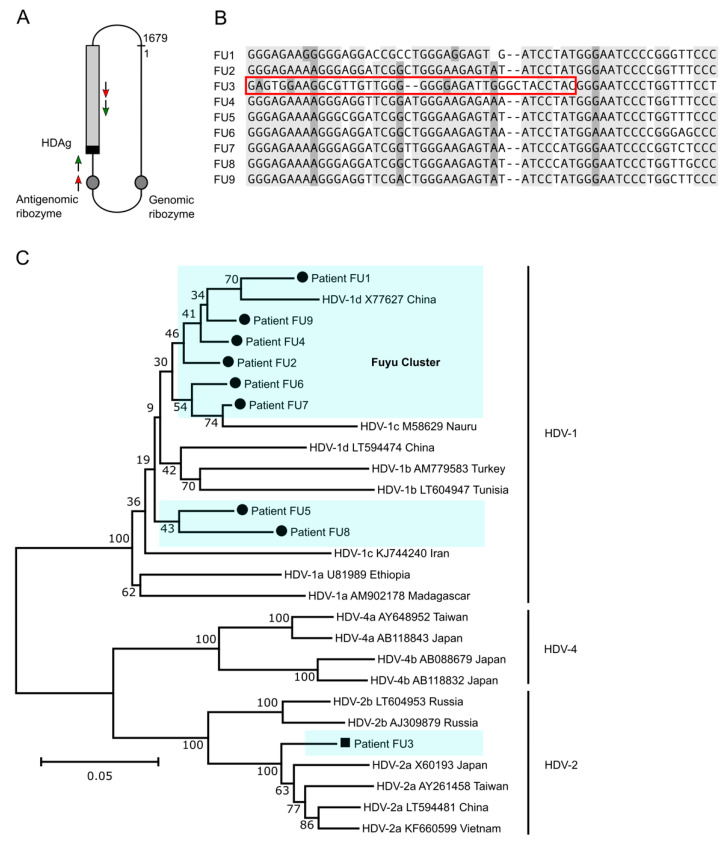
HDV genotyping in a Chinese high-risk population (Fuyu, Jilin province) known for abundant needle sharing during IVDU. (**A**) Scheme of primer binding sites for PCR and Sanger sequencing in the HDV genome. Purified viral RNA from patient serum originating from a high-risk population in the remote northeastern Chinese town, Fuyu (Jilin province), was reverse transcribed using the SuperScript IV reverse transcriptase (Invitrogen, Carlsbad, USA) and cDNA products were amplified by PCR using customized primers p890 and p1265 (red arrows). PCR products were sent for Sanger sequencing. When no sequence data could be obtained, PCR products were further amplified via nested PCR using an internal primer pair (p928 and p1210, green arrows). Grey rectangle: small HDAg reading frame; black rectangle: 19 amino acid extension in large HDAg; grey circles: genomic and antigenomic ribozymes. (**B**) Alignment of nine sequencing results. Light gray: highly conserved; dark grey: conserved showing similarity; white: variable. (**C**) Phylogenetic tree of HDV genomes in nine patients from Fuyu and reference strains. Numbers on the branches represent bootstrap percentages after 1000 replications. The scale bar corresponds to a phylogenetic distance of 0.05 nucleotide substitutions per site.

To determine the origin of HDV strains in Fuyu, we analyzed the sequences from nine HCV-HBV-HDV triple-infected patients. HDV RNA was reverse transcribed and amplified using nested PCR to enhance sensitivity and specificity (Figure 5A) and genomes were sequenced and aligned. Eight of the nine HDV genomes (FU1–2 and FU4–9) were highly conserved and clustered. A single genome (FU3) showed lower homology in the HDAg-encoding region, suggesting an unrelated transmission event (Figure 5B). We further compared the Fuyu HDV genomes with the worldwide occurring genotype 1 viruses (HDV-1) as well as with the eastern Asian circulating genotype 2 and 4 isolates (HDV-2/4). Genomes FU1–2 and FU4–9 were part of the HDV-1 clade, and six of them formed a direct cluster, indicating very close transmission. FU3 was a genetically unique HDV-2a genome and related to strains previously found in China, Japan, and Vietnam (Figure 5C). These findings suggest that at least two distinct HDV viruses were imported to Fuyu, forming a more diverse HDV ecology. Next, we analyzed sequences of HDV-positive patients from mixed populations in Inner Mongolia (27 samples) and Xinjiang (5 samples). All genomes were part of the HDV-1 clade, highly conserved, and formed a wider cluster with the HDV-1 genomes from Fuyu (Figure S1). Our analysis thus indicates close phylogenetic relationships of HDV strains in different regions of China.

## 4. Discussion

HDV presents a significant health burden that may be underestimated. As HDV requires the helper function of HBV and symptoms of HDV infection resemble HBV-associated hepatic disease, international guidelines recommend HDV screening for chronic HBV patients [6,7,8]. However, these guidelines are not strictly followed in the clinical routine. In a university hospital in Germany (Heidelberg), only 47.0% of HBsAg-positive patients received follow-up testing for anti-HDV-Ab between 2014 and 2019. Consequently, 16 anti-HDV-Ab-positive patients were missed, exemplifying an insufficient awareness for HDV among medical staff and the limited availability of standardized procedures for the follow up of HBsAg-positive patients. This should be improved considering the availability of novel antiviral therapies. In many countries including China, HDV diagnostics are not regularly integrated into clinical care due to the limited availability of testing devices. Using the HDV rapid test, we were able to screen a large population of 3419 HBsAg-positive patients from multiple Chinese metropolitan hospitals and from the remote Chinese town of Fuyu. We identified 120 individuals with serological markers for an HDV infection. These findings highlight the suitability of a rapid test for anti-HDV-Ab detection to identify infected patients in settings where lab-based testing is not available.

Insufficient testing for HDV is the major reason for the limited availability of epidemiological data worldwide. Our study shows that 5.3% of HBsAg carriers are co-infected with HDV in a university hospital setting in Heidelberg (Germany). This seroprevalence is slightly lower than previous results from Southwest Germany [42], possibly due to the lower sensitivity of lateral flow assays compared to ELISAs. Other studies indicated a lower seroprevalence in patients from non-clinical contexts, suggesting a potential sample collection bias [43]. Re-testing all samples with a commercial ELISA would help to further eliminate false reactions from the HDV rapid test. HDV seroprevalence estimates in China vary due to non-standardized testing procedures in inhomogeneous patient cohorts. In this study, we found that HDV is not endemic in China but occurs in geographical hotspots and in a high-risk population. The HDV seroprevalence in Inner Mongolia was considerably lower than that reported for Mongolia (13.9% vs. >50%) [18]. Mongolia is known for high HDV endemicity and HDV likely entered China via immigration without extensively spreading. A similar trend can also be observed for Xinjiang province, being geographically close to several central Asian countries with high HDV endemicity. In Uzbekistan, the HDV prevalence was recently shown to be over 80% in cirrhotic patients [21], while in Xinjiang, only 3.9% of a mixed population was co-infected with HDV in our study.

Surprisingly, we found that 16.1% of HBsAg carriers in the remote northeastern Chinese town of Fuyu are HDV-infected, whereas no HDV was found in samples from the nearby provincial capital Changchun (Jilin province). Fuyu was previously shown to be highly endemic for HCV (40%) due to abundant needle sharing for drug injections at social events. The HBV prevalence in this region was 6% [34]. In our study cohort, we observed a significantly higher number of anti-HDV-Ab-positives in HBV-HCV co-infected compared to HBV mono-infected patients, indicating a similar route of transmission for HDV and HCV. Consistently, our sequencing results show that HDV in HCV-HBV-HDV triple-infected patients from Fuyu forms a cluster in the HDV-1 clade, while the presence of a single genotype 2a strain indicates that HDV entered the population via another transmission event. Since no HDV was found in 2528 patients from other provinces, we strongly hypothesize that HDV transmission in China is mostly limited to geographic hotspots and high-risk populations. The absence of HDV in other Chinese metropolitan populations was consistent with early findings of another German-Chinese research collaboration [24].

Recent studies questioned the exclusive role of HBV as a helper virus of HDV [27,31]. To clarify whether HCV can fulfill a helper function for HDV under real life conditions, we took advantage of the known circumstance of HCV spreading via needle sharing in the Chinese town of Fuyu to test for HDV co-transmission. Interestingly, we did not detect anti-HDV-Ab in any of the 365 HBsAg-negative and anti-HBc-negative samples from HCV patients in Fuyu, strongly opposing the hypothesis that HDV transmission associates with HCV in humans. In agreement, two recent studies did not detect HBV-independent HDV in over 2000 HCV patients [32,33]. However, we identified 21/1033 HCV patients with markers of a previously cleared HBV infection that were positive for anti-HDV-Ab. Within this group, two samples were positive for both HDV and HCV RNA but negative for HBV DNA. This indicates that HDV replication can occur in HCV-HBV-HDV triple-infected patients in the absence of measurable HBsAg and HBV DNA. However, such events were rare in our study cohort (2/1033) and HDV RNA levels were low. Our data thus indicates that HCV-supported HDV transmission—although possible in an experimental setting—is unlikely to occur in humans.

## 5. Conclusions

Our study shows that the HDV rapid test can provide access to anti-HDV-Ab screening in large patient cohorts in both developed and developing countries. By using this point-of-care test, we confirmed previous data about the HDV seroprevalence in Germany and found that HDV is not evenly distributed in China but occurs in hotspots associated with geography and IVDU. We also showed that HDV transmission cannot be disseminated by HCV in the absence of HBV markers.

## 6. Patents

The *E. coli*-derived recombinant HDAg with a non-naturally occurring consensus sequence of all eight HDV genotypes is protected by the patent WO2019219840A1.

## Data Availability

The data presented in this study are available on request from the corresponding author. The data are not publicly available due to data privacy and ethical considerations.

## Supplementary Materials

The following are available online at https://www.mdpi.com/article/10.3390/v13091799/s1, Figure S1: Phylogenetic tree of HDV genomes in 41 patients from Inner Mongolia (IM), Xinjiang (XI), Fuyu (FU) and reference strains. Purified viral RNA from patient serum originating from mixed populations in Inner Mongolia (27 samples) and Xinjiang (5 samples) and a high-risk population in the remote Northeastern Chinese town Fuyu (Jilin province, 9 samples) was reverse transcribed using the SuperScript IV reverse transcriptase (Invitrogen, Carlsbad, USA) and cDNA products were amplified by PCR using customized primers p890 and p1265. PCR products were sent for Sanger sequencing. When no sequence data could be obtained, PCR products were further amplified via nested PCR using an internal primer pair (p928 and p1210). Numbers on the branches of the phylogenetic tree represent bootstrap percentages after 1000 replications. The scale bar corresponds to a phylogenetic distance of 0.02 nucleotide substitutions per site.
