# Supplementary material for: HDV Seroprevalence in HBsAg-Positive Patients in China Occurs in Hotspots and Is Not Associated with HCV Mono-Infection
Source: Viruses. 2021 Sep 10;13(9):1799. doi: 10.3390/v13091799 (PMC8473203; doi:10.3390/v13091799)
Supplement: Supplementary file 1 [file viruses-13-01799-s001.zip › viruses-1288674-supplementary.pdf]

viruses

MDPI

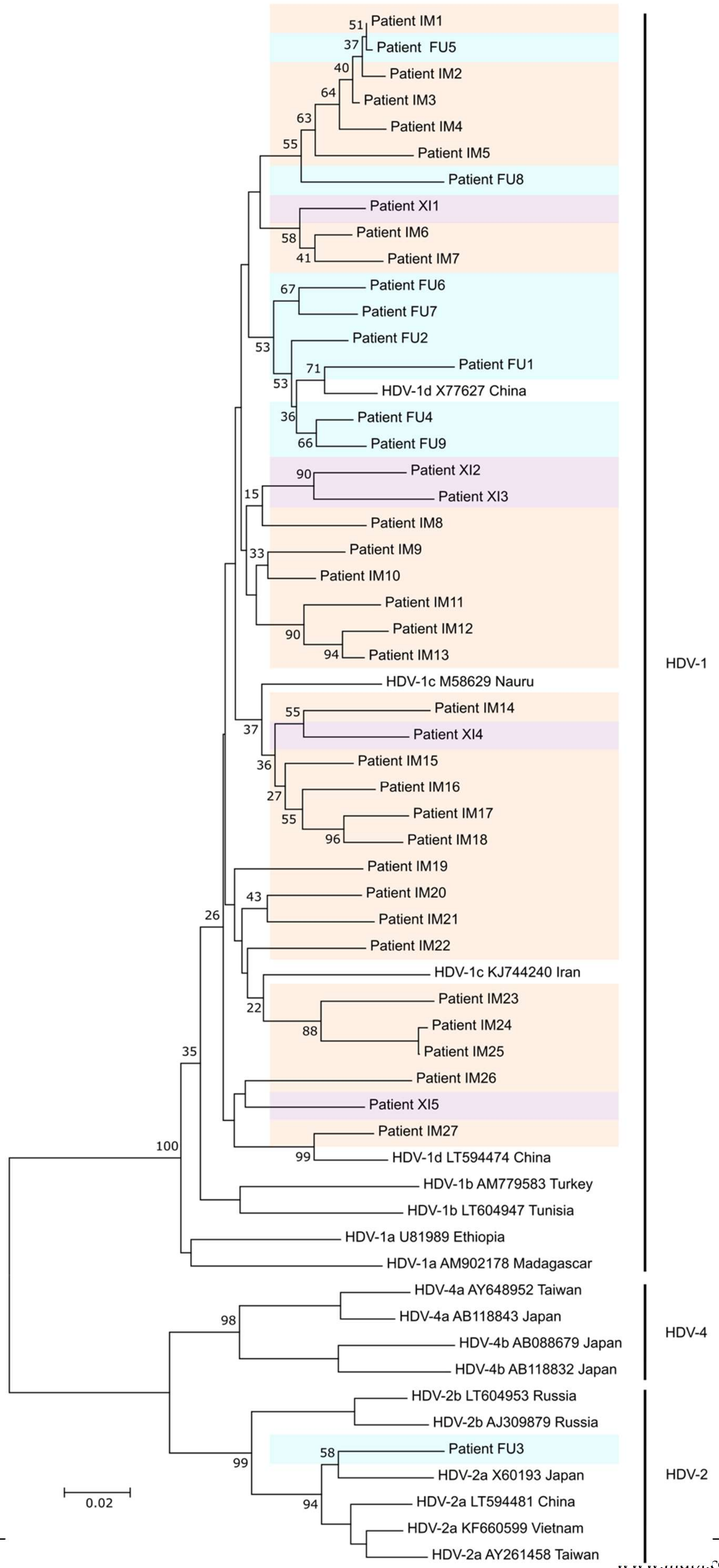

Figure S1. Phylogenetic tree of HDV genomes in 41 patients from Inner Mongolia (IM), Xinjiang (XI), Fuyu (FU) and reference strains. Purified viral RNA from patient serum originating from mixed populations in Inner Mongolia (27 samples) and Xinjiang (5 samples) and a high-risk population in the remote Northeastern Chinese town Fuyu (Jilin province, 9 samples) was reverse transcribed using the SuperScript IV reverse transcriptase (Invitrogen, Carlsbad, USA) and cDNA products were amplified by PCR using customized primers p890 and p1265. PCR products were sent for Sanger sequencing. When no sequence data could be obtained PCR products were further amplified via nested PCR using an internal primer pair (p928 and p1210. Numbers on the branches of the phylogenetic tree represent bootstrap percentages after 1000 replications. The scale bar corresponds to a phylogenetic distance of 0.02 nucleotide substitutions per site.
